# Advancements in Animal Breeding: From Mendelian Genetics to Machine Learning

**DOI:** 10.3390/ijms262311352

**Published:** 2025-11-24

**Authors:** Manjit Panigrahi, Divya Rajawat, Sonali Sonejita Nayak, Anal Bose, Nishu Bharia, Shreyasi Singh, Anurodh Sharma, Triveni Dutt

**Affiliations:** 1 Division of Animal Genetics, Indian Veterinary Research Institute, Izatnagar, Bareilly 243122, Uttar Pradesh, India; simran.singh.sengar3@gmail.com (D.R.);; 2 Livestock Production and Management Section, Indian Veterinary Research Institute, Izatnagar, Bareilly 243122, Uttar Pradesh, India

**Keywords:** breeding, G-BLUP, cattle, genomic prediction, machine learning, Mendelian genetics, QTL mapping, Random Forest

## Abstract

Animal breeding has undergone profound transformations from its origins in phenotypic observation to the integration of genomic and machine learning techniques. This review paper explores the progression of livestock breeding, tracing its roots to the domestication of animals during the Neolithic Revolution. Gregor Mendel’s foundational work with pea plants established key principles of Mendelian genetics, which initially focused on discrete qualitative traits. However, the advancement of quantitative genetics has shifted the focus to continuous traits, such as body weight and milk yield, which are influenced by multiple genes. QTL mapping revolutionized breeding by shifting from phenotype- to genotype-based selection, enhancing accuracy through genomic predictions like GEBV under GBLUP. The strongest QTL associations on chromosome 18 linked local GEBV with *FUK* and *DDX19B* expression. In recent years, machine learning and artificial intelligence have transformed genomic prediction into livestock breeding by efficiently handling high-dimensional data and capturing complex genetic relationships. Notably, a deployed deep learning model achieved an average correlation of up to 0.643 between actual and predicted values. This review highlights the integration of machine learning approaches in animal breeding, showcasing advancements in milk and meat production, and the improvement of disease management through multi-omics strategies. The paper underscores the shift towards innovative methods and their impact on advancing animal breeding practices, offering insights into prospects for enhancing productivity, health, and welfare in livestock.

## 1. Introduction

Animal breeding, the selective breeding for economically significant traits, was traditionally based on phenotypic observations. Livestock breeding has been an essential practice for human societies for thousands of years. The domestication of animals began around 10,000 to 12,000 years ago during the Neolithic Revolution when early humans started to transition from hunting and gathering to agriculture. The first domesticated animals included sheep, goats, cattle, and pigs, selected primarily for traits like docility, productivity, and adaptability to human-controlled environments [1]. With the advent of modern genetics and the Industrial Revolution, livestock breeding underwent significant advancements. Robert Bakewell, the father of modern animal breeding, implemented systematic methods for improving livestock, emphasizing the importance of selecting breeding pairs based on specific traits [2]. This period also saw the rise of breed societies and stud books, which formalized and documented breeding practices [3].

The field of genetics was founded when the first genotype-to-phenotype mapping was presented in Mendel’s pioneering work on peas [4]. Gregor Mendel’s foundational work in genetics delves into how classical breeding methods evolved to include more sophisticated techniques like quantitative genetics and molecular markers. R.A. Fisher (1890–1962), J.B.S. Haldane (1892–1964), and Sewall Wright (1889–1988) established the groundwork for population genetics and scientific animal breeding by creating mathematical models to explain the behavior of genetic frequencies. Nearly 25 years after the structure of DNA was uncovered, the first method for its sequence was developed [5,6]. Meuwissen, Hayes, and Goddard introduced the concept of using a vast number of genotypic markers to predict phenotypes. This approach, known as genomic prediction, involves estimating the combined effects of thousands of markers, typically single nucleotide polymorphisms (SNPs), on a given trait [7]. The BLUP integrated individual and familial records to estimate breeding values (EBV). It provides an optimal way to estimate individuals’ genetic or breeding values based on quantitative genetic principles. From 1990 onward, advances in molecular genetics suggested that incorporating DNA-level information could enhance genetic improvement beyond phenotypic data alone. The use of genetic variation, such as DNA markers, has become widely adopted for predicting genetic merit in animal and plant breeding and is increasingly being used as a prognostic tool for assessing disease risk in human medicine [8].

This led to research into marker-assisted selection (MAS), which involves two key steps: (1) identifying and mapping genes linked to traits of interest, known as quantitative trait loci (QTL); and (2) incorporating QTL information into the BLUP-EBV model [9]. In genome-wide association studies, the number of tests is equal to the number of independent genotyped single nucleotide polymorphisms, often numbering in the tens of thousands in livestock and hundreds of thousands in human genetics [10]. Three key breakthroughs have led to the widespread use of DNA information today: (1) the development of genomic selection (GS) methodology [7]; (2) the discovery of thousands of SNP markers; and (3) the advent of SNP-chip genotyping technologies, which have made large-scale SNP genotyping cost-effective.

The rapid advancement of next-generation sequencing (NGS) has revolutionized biological research by enhancing genomic accessibility, reducing sequencing costs, and accelerating data generation. These technologies have transformed genetic marker discovery, characterization, and application, advancing our understanding of genetics [11]. Genomic selection, which allows for the prediction of an animal’s genetic potential using genome-wide SNP markers, has already been implemented by dairy industries globally and is expected to double genetic gain in milk production and other traits. Following the pioneering work of Meuwissen et al, several whole-genome regression methodologies have been done and evaluated simultaneously to regress phenotypes on whole-genome markers [7]. The range of available methods for implementing whole-genome regression is extensive, including Bayesian regression and genomic best linear unbiased prediction (G-BLUP) from animal breeding, as well as ridge regression, least absolute shrinkage and selection operator (LASSO), the elastic net, support vector regression (SVR), graphical Gaussian models (GGMs), and sure independence screening (SIS) from machine learning [12]. The performance of these methods varies depending on the genetic architecture of the trait. For example, Bayesian methods often outperform BLUP approaches when the trait is less polygenic [8].

Our review aims to explore genetic advancements in animal agriculture to meet the rising global demand for animal products. We highlight key genetic factors influencing health, growth, reproduction, and nutrient utilization, emphasizing cutting-edge research and innovative strategies for sustainable livestock improvement. Additionally, as climate change accelerates, reducing methane emissions from livestock has become a crucial objective [13]. Adopting new livestock technologies, such as genomic selection and advanced breeding methods, will be essential in addressing these challenges. This review highlights the shift from traditional genetic approaches to advanced genomic prediction and machine learning techniques in livestock breeding. We incorporate key scientific advancements over time, detailing major milestones and emerging trends. Additionally, it explores the integration of traditional knowledge with modern technology to enhance livestock productivity and sustainability.

## 2. Pre-Mendelian Era in Early Livestock Breeding

Robert Bakewell (1726–1795) is remembered for setting the pattern of modern animal breeding and is called the founder of animal breeding. However, the early experimental breeders had more philosophy and less science, which changed gradually since the rediscovery of Mendel’s laws and the development of population genetics. While qualitative traits such as polledness and coat color followed typical Mendelian inheritance patterns, most of the other economically important characters varied quantitatively. The early history of population genetics, up to the 1930s, focused on combining the ideas of Darwin’s natural selection with Mendel’s laws of inheritance, leading to the development of the modern evolutionary theory known as Neo-Darwinism. RA Fisher (1890–1962), J.B.S. Haldane (1892–1964), and Sewall Wright (1889–1988), laid the foundation for population genetics and scientific animal breeding by developing mathematical models for the behaviour of genetic frequencies.

**Figure 1:**
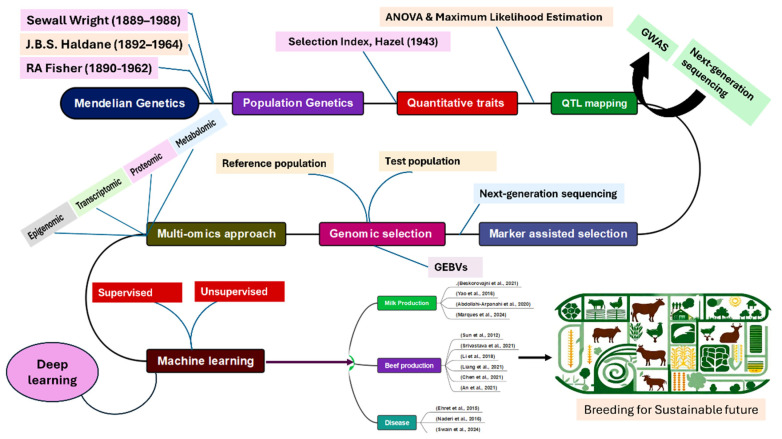
Overview of the trajectory of our review paper, highlighting key developments in the evolution of livestock breeding [15,16,17,18,19,20,21,22,23,24,25,26,27].

Population genetics emerged as a scientific field to study these traits in the early 20th century. The formulation of Hardy–Weinberg law only strengthened Mendelism. In his revolutionary paper, “The correlation between relatives on the supposition of Mendelian inheritance”, Fisher showed how Mendelian genetics could explain the patterns of correlations among relatives in quantitative traits, on the hypothesis that many different genetic as well as non-genetic factors contribute to such quantitative variation [14]. He introduced the mathematical methods that allow the partition of variance into different causal components and thus help in predicting the outcomes of breeding programs. In his series of papers, “Systems of Mating” (1921a–e), Wright applied the ’path coefficient method’ to inbreeding scenarios and its implications for breeding theory and evolution (particularly effective when applied to definite causal lines and linear relations as in Mendelian inheritance). He also founded the ’Fixation Index’. His works in animal breeding were later popularized by J L Lush. Like Fisher and Wright, Haldane’s work also interpreted Darwin’s theory of evolution in the context of Mendelian genetics. Haldane developed statistical models to study the effect of selection and mutation on gene frequencies. His significant mathematical publications, “Mathematical Theory of natural selection”, and his summarized book, “The Causes of Evolution”, helped found population genetics along with the independent contributions of Fisher and Wright. As Fisher, Haldane, and Wright laid the foundation of population genetics using Mendel’s laws and Darwin’s theory as the groundwork, there were several broad gates open now. For example, the development of breeding value, selection index, genetic gain or the breeder’s equation, selection of individuals based on family and pedigree, progeny testing, genomic selection, etc., are the present consequences of the initial efforts made by Mendel and the founders of population genetics. Figure 1 presents an overview of the trajectory of our review paper, highlighting the key developments and milestones in the evolution of livestock breeding from Mendelian principles to modern machine learning techniques.

## 3. Quantitative Trait Loci Mapping and Its Applications in Livestock

**Figure 2:**
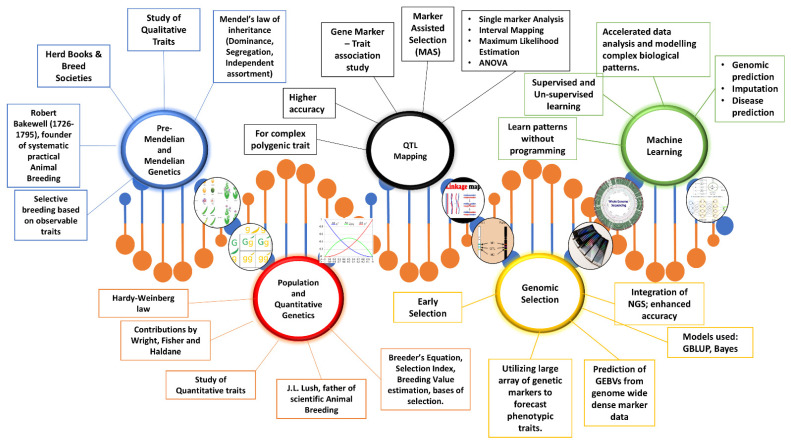
Pictorial representation of studies conducted on animal breeding.

While classical genetics could explain simple traits, there was a need to better understand complex quantitative traits for applications in livestock breeding, as economic traits such as production and growth traits are often quantitative and polygenic, showing continuous variation and cannot be explained by simple Mendelian inheritance. These phenotypic variations may result either from a few loci each having large effects, or from many loci each having small effects. These genes are located in QTL, which are regions within the genome associated with specific traits [28]. A significant amount of the phenotypic variation in many quantitative traits can be attributed to a few loci with large effects, while the rest is due to the influence of numerous loci with smaller effects [29,30,31]. QTL Mapping is a process used to identify these regions showing maximum variations by combining the QTL analysis with linkage maps. Essentially, QTL mapping is a gene marker-trait association experiment [32]. The principle behind QTL mapping involves tracking the inheritance of specific chromosomal segments (genetic markers) from parents to offspring and associating them with trait variations. Identifying QTLs associated with a trait allows for a more accurate estimation of an individual’s breeding value (the potential to pass on desirable traits) through marker-assisted selection (MAS). The process includes key steps such as developing a mapping population, crossbreeding, genotyping, constructing linkage maps, and phenotyping. After obtaining these data, statistical tools like ANOVA and Maximum Likelihood Estimation are used to detect the desired QTLs. They employ various methods to uncover the genetic basis of complex traits by identifying chromosomal regions linked to specific characteristics. One such approach involves the use of genetic markers to pinpoint regions associated with traits, known as marker loci. QTL genotype determination focuses on identifying the specific genotype at the QTL itself. Single Marker Analysis independently examines each marker to evaluate its relationship with the trait, while Interval Mapping assesses the likelihood of a QTL being situated between two markers. Multiple tests are conducted across the genome to discover QTLs, and Maximum Likelihood Estimation is used to statistically estimate the most probable location of a QTL. Additionally, the analysis of multiple QTLs considers the combined effects of several loci to understand their collective influence on the trait. Together, these methods offer a thorough approach to identify and decipher the genetic components that govern complex traits [33]. Figure 2 provides a pictorial representation of significant studies in animal breeding, illustrating their historical progression.

### Applications in Livestock

QTL mapping has emerged as a pivotal tool in livestock breeding, allowing for the identification of genes or markers linked to quantitative traits, which in turn facilitates the early selection of young animals carrying favorable alleles that typically manifest later in life. This early identification significantly boosts the efficiency of genetic improvement through marker-assisted selection (MAS). For instance, in Murrah buffaloes, QTL mapping identified 23 chromosomal regions associated with milk yield, highlighting key meta-QTL regions [34]. In chickens, QTL analysis uncovered at least 30 regions influencing egg production and quality traits [35]. A comprehensive study in pigs over 15 years identified 1675 QTLs related to growth and disease resistance, improving commercial breeding practices [36]. In Angora goats, QTL mapping pinpointed regions linked to conformation traits, marking the first detailed search for genetic variability in these characteristics [37]. Additionally, QTLs on chromosomes 3, 4, and 25 related to wool traits in Merino sheep were identified, including a novel QTL for wool yield on chromosome 25 and QTLs for fleece weight on chromosome 4. These discoveries support the use of MAS to enhance wool production and quality, accelerating genetic improvement and refining breeding strategies [38]. Furthermore, numerous QTLs that are associated with disease resistance in livestock have been identified, notably for mastitis, gastrointestinal nematodes, and other significant diseases in sheep and cattle. Key QTLs include those on OAR1, OAR3, OAR6, and OAR20 in sheep, and BTA7, BTA10, BTA15, BTA18, BTA21, and BTA23 in cattle [39]. These QTLs are utilized in MAS to breed animals with enhanced disease resistance, leading to improved herd health, reduced antibiotic use, and sustainable livestock production. QTL mapping methods can be also effectively utilized in population genomics to identify specific genes associated with inbreeding depression [40]. Various Quantitative Trait Loci (QTL) have been identified for key livestock traits, including conception rate (QTL #176439), and first-service conception (QTL #212394, #212219). Important dairy traits such as lactation persistency (QTL #179346, #18774), milk fat yield (QTL #175862), and milk protein yield (QTL #176255) have also been mapped. A valuable resource for QTL-related data is the Animal QTLdb, which hosts 220,401 QTL, SNP association, and eQTL data linking phenotype to genotype for 2210 traits. In addition, the database provides 23,552 correlations for 866 traits and 4273 heritability data points on 1069 traits in CorrDB. Such comprehensive databases facilitate access to critical genetic information, aiding breeding programs across species [41].

## 4. 1000 Genomes Projects in Livestock

The 1000 Genomes Project in animals, such as the 1000 Bull Genomes Project, are crucial for advancing genomic research in animal breeding, disease resistance, and conservation. By providing large, diverse datasets of high-quality reference genomes, these projects enable more accurate imputation of genetic variants in animals genotyped with SNP arrays, improving genomic predictions for traits like milk yield and disease resistance. They also aid in identifying causal mutations, facilitating targeted genetic selection. Furthermore, these projects enhance genetic diversity knowledge, support precision livestock farming, and promote global collaboration, ultimately contributing to more efficient, sustainable breeding practices and improving animal welfare.

The 1000 Bull Genomes Project is an international collaborative initiative focused on sequencing and analyzing the genomes of over 1000 cattle from various breeds. Launched in 2012, the project aims to provide a comprehensive reference for genetic diversity in cattle, emphasizing traits critical for agriculture, including milk production, disease resistance, fertility, and meat quality [42]. The number of cattle breeds with whole-genome sequences in the 1000 Bull Genomes Project has expanded dramatically, from just 2 in 2012 to 121 today. So far, 84 million single-nucleotide polymorphisms (SNPs) and 2.5 million small insertion deletions have been identified in the collection. Using the sequence data from this project, Boitard et al applied two distinct approaches to detect significant signals of positive selection: a within-population approach to identify selective sweeps, and a population-differentiation approach to capture soft or incomplete sweeps [43]. Their findings confirmed several well-known loci associated with breed-defining and trait-associated characteristics, including MC1R and KIT (coat color and pattern), GHR (growth and milk production), *PLAG1* (stature and age at puberty onset), and NCAPG/LCORL (stature). Additionally, they discovered new loci, such as *ARL15, PRLR, CYP19A1,* and *PPM1L,* highlighting the project’s role in uncovering previously unidentified genetic markers. The availability of the 1000 Bull Genomes data has accelerated the identification of genetic defects and facilitated the detection of causative mutations for complex traits. However, with the rapid growth in both the number of sequence variants and the animals with imputed sequence data, there is a pressing need for more computationally efficient methods to analyze this expanding dataset.

Similar studies have been conducted in other livestock species as well. The 1000 Chinese Indigenous Pig Genomes Project provided a valuable genomic resource, enabling improved imputation performance and facilitating studies on genomic architecture and complex traits in pigs [44]. Key findings, including the detection of ancient admixture events and associations with high-altitude adaptation (13 kb region in the THSD7A) and body size traits (0.47 Mb region on chromosome 7), have been identified. The VarGoats project by Denoyelle et al comprises a comprehensive dataset of 1159 whole-genome sequences, providing valuable insights into the global genetic diversity of *Capra hircus* [45]. Similarly, Fan et al. reported in 2004 that the Duck 1000 Genome Project identified genetic loci associated with key traits in ducks, including growth (e.g., *IGF2BP1* for body size), color (e.g., *MITF* and *MC1R* for white and black plumage), and meat quality (e.g., *TASP1* and *MAGI3* for muscle characteristics), along with metabolite traits linked to genes such as *TMEM189*, *ACBD5*, and GADL1 [46].

The first phase of the 1000BGP analyzed WGS data from 140 buffaloes, identifying 41.6 million SNPs and revealing distinct genetic differentiation between swamp and river buffaloes [47]. About 13 million SNPs with MAF > 1% were shared, while others were specific to each type. The study also identified polymorphisms linked to milk production and reproductive traits, enhancing our understanding of buffalo genomics for future studies on complex traits. Bouwman et al analyzed cattle data to compare the genetic architecture of stature across species, including humans and dogs [48]. They conducted a meta-analysis on stature using data from 58,265 cattle across 17 populations, incorporating 25.4 million imputed whole-genome sequence variants. A significant overlap in stature-related loci among cattle, humans, and dogs suggests that a shared set of genes regulates body size across mammals. FLK and hapFLK tests were utilized in this research to identify enriched SNPs along with the effective genes in the cattle genome through GWAS-based enrichment analysis.

### 4.1. Functional Annotation of Animal Genomes (FAANG) Consortium

The Functional Annotation of Animal Genomes (FAANG) project, launched in 2015, is a global initiative focused on creating comprehensive functional annotations of animal genomes, particularly for livestock species. By mapping functional elements like regulatory regions and gene expression patterns, FAANG aims to enhance genetic selection, breeding strategies, and animal health [49]. The project fosters global collaboration, data sharing, and high-quality data standards to improve productivity, sustainability, and animal welfare. The FAANG project includes several task forces focused on specific aspects of animal genomics. FAANGCompGen works on comparative genomics and genome assembly for cross-species analysis. FAANGPrediction integrates FAANG data to predict phenotypes and improve breeding evaluations. FAANGSingleCell explores gene expression at the single-cell level, unraveling regulatory complexity. FarmGTEx links genetic variation to tissue-specific gene expression to study traits like growth and disease resistance. HTP-DS enhances the management of phenotypic data from high-throughput technologies. metaFAIR ensures FAANG data follow FAIR principles for better accessibility and global collaboration. Additionally, the highly annotated genomes produced by FAANG enable the definition of evolutionary conservation across species, supporting pan genomic analyses [49,50,51].

Young et al constructed a gene expression atlas from 220 tissue and cell samples across three river buffalo breeds (Mediterranean, Pandharpuri, and Bhadawari). This effort yielded over 21 billion raw sequence reads mapping to 18,730 unique genes, facilitating the annotation of the buffalo genome [52]. Regulatory elements in pigs were cataloged using 223 epigenomic and transcriptomic datasets across 14 tissues [53]. Chromatin states were annotated, revealing tissue-specific regulatory activities. Variants linked to traits and evolution were enriched in promoters and enhancers, with distinct regulatory selection observed between Asian and European domestication. Compared to humans and mice, porcine regulatory elements showed higher DNA sequence conservation. Kern et al analyzed data from eight tissues in chicken (Gallus gallus), pig (Sus scrofa), and cattle (Bos taurus) alongside human and mouse ENCODE data. Core regulatory elements and tissue-specific transcription factor activity were found to be conserved across species [54]. Goszczynski et al enhanced the bovine genome’s functional annotation by identifying transcription start sites (TSSs) using the RAMPAGE method across various tissues. The findings include novel TSSs for protein-coding and lncRNA genes, validated via experimental and in silico methods [55]. A promoter activity atlas was created, revealing tissue-specific promoter usage, notably in brain and testicle tissues. Coexpression networks identified tissue-enriched transcription factors and alternate loci usage. This comprehensive TSS annotation aligns bovine genome resources with human and mouse resources, providing a foundation for advanced gene regulation studies and improved livestock genomics.

### 4.2. Transcriptome-Wide Association Studies (TWAS) in Livestock

The genetic variation of key livestock traits is crucial for genetic improvement. Genome-wide association studies (GWAS) have identified many loci associated with complex traits, but most of these are in non-coding regions with unclear functions. To address this, expression quantitative trait loci (eQTL) and transcriptome-wide association studies (TWAS) are used to identify pivotal expression-trait associations, which have proven successful in cattle. Three strategies were employed to identify candidate genes affecting the productivity of Huaxi cattle: GWAS, TWAS, and an ensemble approach combining the two. The study focused on longissimus dorsi muscle (LDM) weight as the phenotype and analyzed candidate genes using Gene Ontology (GO) and Kyoto Encyclopedia of Genes and Genomes (KEGG) analyses to enhance Huaxi cattle breeding. Fisher’s combined test was used to integrate the results from GWAS and TWAS to identify the most significant genes. For gene functional analysis, Gene Ontology (GO) and KEGG pathway analyses were applied to understand the roles of the candidate genes involved in LDM weight variation, providing insights for future genetic improvements in Huaxi cattle [56].

### 4.3. Mendelian Randomization (MR) Methodology

The MR method is applicable to both human and animal studies, emphasizing the importance of model checking to ensure valid causal inferences. With the growing availability of large-scale GWAS summary data and advanced software, MR and TWAS are increasingly used to explore causal relationships between complex traits, such as gene expression and other traits. These methods use genetic variants as instrumental variables (IVs). This method shows higher statistical power than existing methods and highlights frequent violations of IV assumptions in TWAS using, for example, GWAS data from schizophrenia, Alzheimer’s disease, and blood lipids. The study emphasizes the need for model checking in MR and TWAS, and the proposed method could be valuable for this purpose [57].

### 4.4. Integrative Genomic Analyses Using Phenome-Wide Association Studies (PheWAS)

PheWAS is used to explore the association between genetic variants (often single nucleotide polymorphisms, or SNPs) and a broad range of phenotypes (observable traits or conditions). GWAS typically investigate the association between genetic variants and a single trait or disease. PheWAS take a broader perspective by assessing how genetic variants might influence multiple traits simultaneously. PheWAS was employed to explore the phenotypic consequences of genes identified through TWAS, a methodology that can be translated to livestock studies [58]. PigBiobank Resource provides insights into the genetic and biological mechanisms underlying complex traits in pigs, utilizing approaches like TWAS and PheWAS to enhance understanding of trait associations [59].

### 4.5. FarmGTEx

FarmGTEx is an extension of the Genotype-Tissue Expression (GTEx) project, which focuses on understanding how genetic variation influences gene expression across a wide variety of tissues. Specifically, FarmGTEx aims to integrate genetic data with gene expression profiles to better understand the biology of agricultural and livestock species, such as cattle, pigs, chickens, and others. FarmGTEx aims to map how genetic variants affect gene expression in agricultural animals. The primary focus of FarmGTEx is to enhance breeding programs by providing insights into genetic influences on traits of economic importance in livestock farming. FarmGTEx extends the concepts of GTEx by potentially comparing how genetic variants affect gene expression in livestock versus humans, providing a unique opportunity for comparative genomics [11,56].

## 5. Genomic Selection (GS)

### 5.1. Overview of the Transition from Phenotype-Based Selection to Genotype-Based Selection

In the last ten years, livestock breeding has been shifting in the direction of genomic selection. G-BLUP is considered the method of choice for the estimation of breeding values in purebred breeding schemes [60]. This approach is associated with the quantitative trait locus associated with the particular phenotypic trait of interest. To put it in a nutshell, in GS, a reference population is genotyped and then evaluated for a phenotype, such that the effects of SNPs against the trait are estimated, and then the candidates for selection are genotyped and their genomic data are combined with the estimated SNP effects to predict GEBVs. In pure breeds, this has been implemented as one of the cores of the system: reference population, genotyped, and phenotype animals. The phenotypes are either performance records of the animal or de-regressed conventional breeding values. This reference population is used to predict marker effects, which was required as an initial step for genomic breeding value prediction of genotyped selection candidates. Reliability depends on the size of the reference population, the effective number of chromosome segments, and the method used for the prediction of marker effects [61]. Unlike traditional BLUP-based EBVs, the GS approach does not rely on pedigree records or require the selection candidates to have trait measurements. In contrast, traditional BLUP estimates EBVs based on phenotypic data and family relationships derived from animal pedigrees.

Predictions were made using both linear and nonlinear systems of equations. The linear predictions assumed genetic variation arose from a very large number of markers, all contributing equally, expecting no influence of any major gene. In contrast, nonlinear predictions or Bayesian predictions incorporated the assumption that the prior distribution of marker or QTL effects was non-normal [62]. Multi-breed genomic predictions offer the potential to enhance the accuracy of genomic predictions by leveraging information from multiple breeds. This approach may be particularly beneficial when dealing with crossbred or composite animals. However, multi-breed genomic evaluations are more complex than single-breed evaluations due to the increased diversity of haplotypes present at any given genomic location in multi-breed datasets [63]. Genomic prediction involves utilizing a large array of genetic markers to forecast phenotypic traits [7]. There are two primary methods for estimating marker effects. The first method approximates a traditional infinitesimal model, assuming that all markers, typically single nucleotide polymorphisms (SNPs), contribute a non-zero value to genetic variance and that SNP effects follow a normal distribution. The second method employs nonlinear techniques that focus on specific genomic regions and permit marker effects to derive from distributions other than the Gaussian. According to Wiggans et al and Lund et al, accuracy in genomic value predictions for production traits exceeded 0.8, more than 0.7 for fertility, longevity, and other characteristics like the somatic cell count, in the field of dairy cattle [64,65]. Traditional genetic improvement has typically depended on recording each animal’s phenotype and using pedigree information to estimate its breeding value (BV), commonly employing the statistical method known as BLUP [66].

Methods of Bayesian genomic predictions were designed to take into consideration all the parallel genotyped markers when making a prediction on breeding values for quantitative traits. These methods accommodate variations in the genetic architecture, specifically the distribution of marker effects across different traits, enabling more accurate predictions. Bayes-A assigns each marker (haplotype) a normal prior distribution with its variance. In contrast, the Bayesian variable selection (BVS) model, Bayes-B, is like Bayes-A but includes a prespecified prior proportion, π, where a certain percentage of genetic markers (haplotypes) are assumed to have zero effects [67]. In G-BLUP, all markers are assumed to explain an equal amount of variance; with the Bayes C approach, markers can explain different quantities of variation, and in the case of oligogenic traits, a small number of markers are assigned to have an effect, and many markers to have no effect [68,69]. Bayes C is a modification of the original Bayes B approach by Meuwissen et al in which the proportion of SNP with zero effects is estimated from the data [7,70]. BayesCπ-type methodology assumes that prior SNP effects are either zero or normally distributed (Stock et al., 2020) [60]. The linear methods assume that all markers have non-zero effects, which in general are considered to be normally distributed. Examples of linear methods include the ridge regression best linear unbiased prediction (RRBLUP) and G-BLUP [62,71,72].

### 5.2. Genomic Selection

In genomic selection, Genomic Estimated Breeding Values (GEBV) under the GBLUP approach are estimated using phenotypic and genomic relationships derived from genome-wide dense marker data. GBLUP closely resembles the traditional BLUP method, but with genomic relationships replacing pedigree relationships. This approach offers a practical advantage, as existing BLUP software (https://www.blup.in/) can be used with only the replacement of pedigree data by genomic relationships. Unlike SNP-BLUP, which requires the estimation of effects for about 50,000 SNPs and thus involves solving 50,000 equations, GBLUP requires estimating GEBVs for N animals, where N is typically less than 50,000 genotyped animals, making it computationally more efficient.

A key assumption in both SNP-BLUP and GBLUP is that SNP effects follow a normal distribution with a common variance across SNPs. The SNP-BLUP model assumes normally distributed SNP effects. Other models, like BayesC, BayesB, and BayesR, introduce different distributions for SNP effects [7,73]. BayesC uses normal distributions with constant variance, similar to SNP-BLUP. BayesB applies a t-distribution, allowing some SNPs to have larger effects, while BayesR assumes a mixture of normal distributions, enabling certain SNPs to have very large effects. The single-step GBLUP (ssGBLUP) method integrates phenotypic, pedigree, and genomic data using a combined matrix H, allowing for the prediction of genomic merit values for both genotyped and non-genotyped individuals. This approach [74,75,76] leverages all available information, significantly improving the accuracy and efficiency of genetic selection programs across various species [76,77,78].

### 5.3. Implementation in Livestock Breeding

Goddard (2010) suggested a long-term optimum response strategy that, for QTL, which is initially common and with large effects, reduces selection pressure compared to selection based solely on EBVs [61]. When using very high-density SNP genotyping along with the Bayes B method to estimate SNP effects, only SNPs in close linkage disequilibrium (LD) with the QTL exhibit estimated effects with accuracy that remains consistent over time, as the LD persists. Inbreeding reduces long-term response as well, and results in inbreeding depression. Traditional selection can consider the balance between maximizing the EBV of selected animals and minimizing long-term inbreeding by optimizing each animal’s contribution to the next generation [79,80]. This approach can be extended to genomic selection by using the relationship matrix estimated from SNPs [81]. To achieve large-scale implementation of genomic selection, refining field management is crucial for enhancing heritability estimation and prediction accuracy. Additionally, optimizing GS models by incorporating genotype-by-environment interactions and non-additive effects, while reducing costs, can further improve efficiency. Integrating GS with other breeding technologies and platforms can accelerate genetic improvement and maximize genetic gain. Furthermore, fostering an open-source breeding network and adopting trans disciplinary approaches will be instrumental in advancing breeding efficiency.

## 6. Molecular Genetics Advances in Terms of Animal Breeding

### 6.1. Advances in Sequencing Technologies

The rapid evolution of sequencing technologies has had a profound impact on livestock genetics, transforming the landscape of genomic research in farm animals. High-throughput sequencing platforms, particularly those based on third-generation sequencing (TGS), have revolutionized the study of genomic diversity, epigenetics, metagenomics, and the identification of single nucleotide polymorphisms (SNPs) and copy number variations (CNVs). These advancements have accelerated the understanding of biological processes and evolutionary mechanisms in livestock species, including cattle, swine, and horses, which are not only economically valuable but also serve as essential models for biomedical research [82].

A high-quality genome assembly serves as the cornerstone of genetic research, providing an accurate and comprehensive representation of an organism’s genetic architecture. For genetic improvement programs, robust genome assemblies are essential as they enable better characterization of genes, regulatory elements, and genetic variation, thus informing breeding strategies. The advent of third-generation sequencing (TGS) and long-range sequencing technologies, such as Hi-C and Strand-Seq, have led to the development of platinum-quality telomere-to-telomere genome assemblies [83]. These technologies have transformed genome assembly by offering long, highly accurate reads, which significantly reduce the assembly gaps and errors commonly encountered with short-read technologies. Third-generation sequencing technologies, such as those developed by Pacific Biosciences and Oxford Nanopore, offer significant advancements with read lengths exceeding 10 kilobases—far surpassing both Sanger and short-read methods [84]. These long-read sequencing technologies effectively address challenges associated with short-read sequencing, such as difficulties in detecting genome-wide repeats and structural variants. Unlike second-generation methods, third-generation sequencing requires minimal library preparation and directly targets unfragmented DNA molecules in real time. The primary limitation has been the accuracy of these reads, though this has continually improved, particularly with advances in software analysis [85].

Alongside advancements in sequencing technologies, computational tools and algorithms have evolved at a remarkable pace, facilitating more efficient data analysis and interpretation. These developments have enabled the emergence of the concept of the pangenome, which integrates genetic variation across multiple individuals of a species, providing a comprehensive view of the genetic landscape. Recent developments in genomics have highlighted several limitations associated with using a single reference genome, such as reference bias in variant calling, inaccurate structural variant (SV) detection, incomplete RNA-Seq analyses, and errors in genotyping complex genomic regions [86,87,88]. These issues arise because a single reference genome cannot fully capture the genetic diversity present across different individuals or populations. Pangenome studies offer a solution to these challenges by providing a more comprehensive representation of genetic variation. By overcoming the biases inherent in single-reference genomes, pangenomes enable more accurate detection of variants, SVs, and complex genomic features, ultimately improving the precision of genetic studies [89]. Methods for pangenome construction, such as de novo assembly, Map-to-Pan, and iterative mapping, have made it possible to detect previously hidden genetic diversity, including large-scale SVs and novel functional loci. In livestock species such as cattle [90], sheep [91], pigs [91] and chickens [92] pangenome studies have uncovered important insights into domestication, adaptation, and phenotypic variation. These insights are crucial for improving breeding strategies and identifying genetic markers associated with traits like disease resistance, growth, and fertility.

### 6.2. Multi-Omics Approaches

In the context of livestock breeding, integrating multi-omics data on breeding populations and individuals enables a deeper understanding of the regulatory networks that control gene expression and phenotype formation across different populations, breeds, and species. By merging these diverse datasets, researchers can construct more accurate multi-omics regulatory breeding models that account for the interplay between various molecular layers and their impact on important traits. The decline in sequencing costs has also accelerated functional genomic research in major livestock species, facilitating the exploration of molecular mechanisms and key genes responsible for phenotypic variations in economically important traits [93]. Transcriptomics has been widely used to identify candidate genes associated with economic traits in livestock, providing critical insights into RNA transcription. Numerous candidate messenger RNAs (mRNAs), microRNAs (miRNAs) and long non-coding RNAs (lncRNAs) that influence major economic traits have been identified. Proteomics technology has advanced the identification of proteins linked to meat quality and muscle development, particularly those differentially expressed during various developmental stages [94,95]. These findings lay the groundwork for improving meat production and quality through targeted breeding strategies [96]. Epigenetic changes, like histone methylation, have been widely researched in livestock. For instance, research on bovine peripheral blood lymphocytes revealed that H3K27me3 methylation modulates gene expression across the entire genome [97]. Functional genomics approaches enable the exploration of molecular mechanisms that drive phenotype development and the intricate regulatory networks in livestock. Through the use of genomic analysis techniques, contemporary systems biology has revealed genomic variations across multiple levels, such as sequence, structure, epigenetic modifications, and transcription, and their impact on phenotype evolution [98].

Traditional GWAS approaches are now being supplemented with multi-omics-based GWAS. The integration of multi-omics approaches with GWAS enhances the identification of genetic markers associated with complex traits by linking molecular variations across multiple biological levels. For example, transcriptome-wide association studies (TWAS) use gene expression data to refine GWAS hits, while epigenome-wide studies (EWAS) examine how methylation patterns influence phenotypes. Similarly, metabolome-based GWAS (mGWAS) assesses the relationship between SNPs and metabolites, providing insights into intermediary pathways connecting genetic variation to phenotypic outcomes. The integration of multi-omics GWAS into GS revolutionizes breeding by improving the accuracy of GEBVs. Multi-omics data enrich GS models by incorporating non-genetic factors such as transcriptomic profiles and epigenetic marks that influence heritable traits. Moreover, mGWAS contributes intermediary metabolite data, directly linking genotype to economically significant traits like feed efficiency or disease resistance, which are challenging to evaluate. This combination of multi-omics GWAS and GS heralds a new era of precision breeding, fostering sustainable improvements in livestock productivity and welfare while addressing challenges like environmental adaptability and complex trait heritability.

Systems biology approaches have further elucidated the molecular mechanisms governing intergenic interactions, regulatory networks, and the interplay between various omics levels, all of which contribute to phenotype formation [99]. Understanding these gene networks is essential for analyzing the genetic architecture of complex phenotypes. The development of cost-effective high-throughput sequencing technologies and genotyping platforms has made it easier to study livestock gene functions [100]. As these costs continue to decline, large-scale genome-wide sequencing for livestock breeding will become increasingly feasible. Additionally, integrating multi-omics data from diverse breeding populations will significantly enhance breeding accuracy, accelerate breeding progress, and reduce costs.

## 7. Roles of Emerging eRNAs in Animal Breeding

Enhancer RNAs (eRNAs), a class of non-coding RNAs transcribed from active enhancers, have emerged as key regulators of gene expression influencing various traits in livestock. Unlike other regulatory RNAs, eRNAs facilitate enhancer-promoter interactions, promoting tissue-specific gene expression crucial for animal breeding [101]. They are transcribed bidirectionally, typically unstable, and closely associated with active enhancer states marked by histone modifications like H3K27ac and transcription factor (TF) binding [102,103]. Though initially considered transcriptional noise, eRNAs are now recognized for their role in chromatin looping, TF stabilization, and co-activator recruitment [15,104]. Their expression is linked to multiple traits, such as body weight regulation through the eRNA OLMALINC, which controls stearoyl-coenzyme A desaturase [105]. TFs also regulate eRNAs, as seen in estrogen receptor 1 (ESR1)-induced eRNAs that maintain transcriptional networks in breast cancer and MyoD enhancer-derived eRNAs that mediate cohesin recruitment during muscle differentiation.

While early research focused on human biology, recent studies have explored eRNAs in livestock, leading to the development of animal-specific databases like AnimalTFDB 3.0 [106], AnimalQTLdb [107], Animal-imputeDB [108] and Animal-eRNAdb, which identified trait-related eRNAs, putative eRNA regulators, putative eRNA target genes, and eRNAs with sequence similarities across different tissues in different species by methodically quantifying the expression of eRNAs using data from 5085 samples from 10 species [101]. The “Pig-eRNAdb” study identified 37,803 eRNAs across 15 pig tissues, linking them to 652 key traits, including muscle growth, fat metabolism, reproduction, and disease resistance. Housekeeping eRNAs (HKeRNAs) played essential roles in gene regulation, chromatin silencing, and immunity. Notably, 81.4% of pig eRNAs were conserved with human eRNAs, underscoring their biomedical relevance. These findings offer valuable markers for precision breeding to enhance livestock productivity and health [109]. In mice, eRNAs influence myogenic differentiation [101,104], while in sheep, they have been associated with growth traits, emphasizing their potential for genetic selection [101]. Hence, eRNAs serve as key biomarkers and regulatory elements in livestock breeding, influencing growth, reproduction, immunity, and metabolic traits. Their association with enhancer-linked SNPs (eSNPs) makes them valuable for genomic selection. As sequencing technologies advance, validating and integrating eRNAs into breeding programs will enhance precision and efficiency, driving sustainable genetic improvements.

## 8. Machine Learning and Artificial Intelligence in Genomic Prediction

### 8.1. Introduction to Machine Learning

Machine learning is generally described as the ability of machines to replicate intelligent human behavior. It serves as a method for implementing AI. In the 1950s, artificial intelligence innovator Arthur Samuel described machine learning as “the field of study that gives computers the ability to learn without explicitly being programmed” [110]. Machine learning has the potential to transform life science research by accelerating data analysis, predicting biological patterns, and modeling complex biological systems. In machine learning, two types of data are used: training and test data. Training data enable the algorithm to learn, while test datasets are utilized to assess its performance and effectiveness for a particular task [111]. Machine learning is perhaps basically categorized into two main categories: supervised and unsupervised learning. Supervised learning relies on labeled datasets, where input data come with corresponding correct outputs (labels). The model learns to map inputs to outputs by minimizing errors through training, whereas unsupervised learning works with unlabeled datasets, where the program must find inherent patterns or structures without explicit guidance. Supervised learning is divided into regression and classification categories. Regression is used when dealing with real-valued output variables, utilizing algorithms such as Simple Linear Regression, Multivariate Regression, Decision Tree Regression, and Lasso Regression. Conversely, classification is used when the output variable is categorical. Common algorithms for this purpose include Random Forest, Decision Tree, Logistic Regression, and Support Vector Machine [112]. Simple Linear Regression and Decision Tree Regression are two popular regression techniques in supervised learning. The unsupervised learning approach uses input data without labeled responses to uncover hidden structures within the data. It encompasses several categories, such as clustering involves grouping similar instances into clusters, with popular algorithms such as K-Means Clustering, Mean-Shift, DBSCAN, Principal Component Analysis (PCA), and Independent Component Analysis (ICA) [113,114].

Within AI, machine learning is a key area that allows machines to learn from data and get better over time without needing specific programming. Deep learning is a subset of machine learning that utilizes artificial neural networks with many layers, commonly referred to as deep neural networks. Deep learning is a more advanced part that uses neural networks, which are algorithms influenced by the human brain. These networks are especially good at tasks like image and speech recognition, forming the core of deep learning and driving progress in AI. Recently, machine learning algorithms, combined with advancements in computational processing power, have generated significant interest in the scientific community. These models are highly versatile and particularly effective at discovering hidden patterns in large, noisy datasets. Examples include image-based data [115] and extensive collections of heterogeneous records [116]. Additionally, these models excel in processing rapidly expanding digital data, driven by advancements in computer vision, natural language processing (NLP), the Internet of Things (IoT), and computer hardware [117].

### 8.2. Integrating Machine Learning in Animal Breeding

With the global population on the rise, animal breeding is increasingly being employed as a sustainable strategy to improve food security. Numerous high-throughput omics technologies have been devised and applied in animal breeding to accelerate genetic improvements and create new breeds with higher productivity and highly efficient to climate change, pests, and diseases. With these advanced technologies, large amounts of data have been generated on the genetic architecture of animals, which can be exploited to manipulate key traits important for breeding improvement. Consequently, in order to effectively analyze huge and complicated datasets, animal breeders are now depending more on high-performance computing, bioinformatics tools, and artificial intelligence (AI), particularly machine learning (ML) techniques [118]. Even while machine learning plays a big part in daily life, the field is still in its infancy when it comes to using ML in animal breeding and production.

In the post-genomic era, animal breeding involves working with extensive high-dimensional datasets, including genomics, epigenomics, transcriptomics, proteomics, and metabolomics. These datasets are often large, complex, and prone to issues like genotyping errors, missing data, batch effects, and biological variability. ML techniques allow breeders to extract meaningful insights from these complex datasets, facilitating the selection of animals with desirable traits. One of the most critical applications of ML in animal breeding is genomic prediction [119]. Traditional statistical methods, like Genomic Best Linear Unbiased Prediction (GBLUP), have long been used to estimate breeding values, but ML techniques, such as Random Forests (RF), Support Vector Machines (SVM), and kernel ridge regression (KRR) are proving to be more effective for capturing nonlinear relationships between genotypes and phenotypes [120]. Deep learning models, including Convolutional Neural Networks (CNNs) and Recurrent Neural Networks (RNNs), have further enhanced genomic prediction by uncovering complex interactions among genetic markers. One such example is the recently developed deepGBLUP framework, which combines the strengths of DL and GBLUP by introducing a novel locally connected layer (LCL) to improve genomic prediction accuracy [121]. Imputation, the process of predicting the missing genotypes, is essential for completing genomic datasets and maximizing the utility of available genetic information. ML methods have emerged as highly effective tools for improving imputation accuracy, surpassing traditional statistical approaches [122].

Additionally, machine learning algorithms can be utilized to predict disease occurrence by integrating genotype data with health records. For instance, ML techniques were applied to address a significant health issue in the intensive dairy industry, specifically the risk of subclinical ketosis [16]. Additionally, training ML models on biological data presents several challenges [123]. For example, when integrating marker data, environmental data, and phenotypic records to predict outcomes, the high variability of the input data can create significant obstacles. To address this, it is crucial to perform a pre-processing step that involves formatting, cleaning, scaling, and normalizing the data. This gear is necessary for optimizing the accuracy and performance of the machine learning model. Marker datasets are typically large and contain a significant amount of noise. Utilizing raw data without preprocessing can result in poor model performance and overfitting [124]. Therefore, performing feature selection is crucial when working with omics data as it reduces data dimensionality by identifying and retaining relevant features while filtering out noise. Various methods can be employed for feature selection, including statistical approaches, correlations, and hypothesis testing. Recently, machine learning models have demonstrated significant effectiveness in feature selection. Widely used ML-based feature selection approaches include selection criteria methods, which combine aspects of both filter and wrapper techniques [125]. These ML-based approaches are particularly useful when handling marker datasets in animal species. Additionally, when training ML models on biological data, it is essential to ensure data quality through preprocessing steps. Furthermore, optimizing model performance involves adjusting hyperparameters and employing regularization techniques. There are several techniques available for this, such as gradient descent, stochastic gradient descent, random search, grid search, Bayesian optimization, and genetic algorithms. Table 1 shows the categories, focus areas, and key findings on the application of machine learning in livestock improvement. Similarly, Table 2 presents a summary of major quantitative trait loci (QTL) discoveries across various livestock species, highlighting key genetic regions associated with economically important traits.

### 8.3. Case Studies on Utilizing Machine Learning Approaches in Cattle Breeding

In recent years, machine learning has seen a surge in successful applications within genomic prediction. This approach is advantageous because it requires fewer assumptions, effectively manages the challenges of high-dimensional data, and offers greater flexibility in capturing complex relationships.

#### 8.3.1. Milk Production

Recent advancements in machine learning have significantly enhanced the predictive capabilities for various traits in dairy cattle, offering promising tools for improving yield, fertility, and overall herd management. Beskorovajni et al demonstrated the power of an ANN model, utilizing the Broyden–Fletcher–Goldfarb–Shanno iterative algorithm, to predict yield and fertility traits in milk cattle [17]. Their model exhibited strong predictive accuracy, with R^2^ values during the training cycle ranging from 0.444 to 0.989. This study highlights the potential of advanced machine learning algorithms in livestock management. In an earlier study, the focus was on optimizing the genomic prediction of residual feed intake in the exotic HF cattle breed [18]. They examined the effect of altering the ratio between animals with self-reported phenotypes and those with measured phenotypes in genomic prediction models. The results showed that the advantages of using self-trained phenotypes decreased with the size of the first training set. For instance, with the increment of training sets, the optimal ratio of self-trained to measured phenotypes decreased, alongside a corresponding reduction in the maximum increase in prediction accuracy (5.9%, 4.1%, and 2.4%, respectively). This suggests that while self-trained phenotypes can enhance prediction accuracy, their utility may be limited as training sets grow larger.

Further advancements were made by Abdollahi-Arpanahi et al., who conducted a comprehensive study on Holstein dairy cattle to predict the sire conception rate (SCR) using various machine learning methods [19],. The predictive performance of deep learning techniques (MLP and CNN), ensemble learning techniques (RF and Gradient Boosting), and parametric techniques (GBLUP and Bayes B) was examined through their analysis of 1170 datasets containing 57,749 SNPs.

The study found that Gradient Boosting achieved the highest predictive correlation (0.36), outperforming Bayes B (0.34), GBLUP (0.33), Random Forest (0.32), CNN (0.29), and MLP (0.26), underscoring the effectiveness of ensemble learning in this context. Building on these findings, a machine learning approach was developed to predict pregnancy in dairy cows by integrating automated activity monitoring (AAM) with on-farm data [20]. The study demonstrated that combining on-farm data, such as health and environmental conditions, with AAM data provided a more accurate prediction of a cow’s pregnancy likelihood. Among the methods employed, the Random Forest model was particularly effective in reducing prediction errors, showcasing the value of integrating diverse data sources for enhanced predictive accuracy in dairy herd management. Together, these studies illustrate the growing role of machine learning in advancing the precision and accuracy of predictions in dairy cattle, paving the way for more informed decision-making and improved animal welfare in the industry.

#### 8.3.2. Beef Production

These efforts span across different breeds and traits, utilizing various genotypic and phenotypic data to enhance livestock management and breeding strategies. A comprehensive analysis was conducted of 3078 registered Angus cattle by a group of researchers. They compared the accuracy of following imputation packagesBeaglev5.5, IMPUTE2v2.3.2, fastPHASEv2.0.4, AlphaImpute2, findhap.f90 [21]. Beagle and Fimpute emerged as the top performers, achieving accuracy values between 0.8677 and 0.9858. To further enhance imputation accuracy, they proposed an AdaBoost-like approach that combines results from multiple independent software packages, setting a precedent for multi-tool integration in genomic studies. Building on the integration of machine learning in livestock management, Srivastava et al focused on predicting key carcass traits in Hanwoo cattle, a breed prized for its marbling quality [22]. Their study utilized phenotypic and genotypic data, including 53,866 SNPs from 7324 cattle, to assess the accuracy of predictions of deep learning techniques. The findings revealed that XGB provided the highest predictive correlation for carcass weight (CWT) and marbling score (MS), while GBLUP led in predicting backfat thickness (BFT) and eye muscle area (EMA), underscoring the nuanced performance of different models across traits. In 2018, Li et al extended the application of machine learning to Brahman beef cattle, analyzing 2093 samples with 40,184 SNPs to evaluate body weight (BW) [15]. They employed Random Forest (RF), Gradient Boosting Machine (GBM), and XGBoost to identify top-ranked SNPs, which were subsequently used to construct genomic relationship matrices for estimating genomic breeding values. Their work highlighted the utility of machine learning in refining genomic selection by focusing on key genetic markers. Similarly, in 2021, Liang and colleagues investigated ensemble learning models to forecast genomic values for three economically important traits in Simmental beef cattle [23]. Utilizing data from 1217 samples with 671,900 SNPs, they employed Adaboost.RT (combined with SVR). Their findings indicated that these machine learning approaches surpassed GBLUP, achieving average accuracy improvements between 5.4% and 14.9%, highlighting the potential of advanced models to improve prediction accuracy in genomic selection. Machine learning methods were applied to a different aspect of cattle management, focusing on Nellore beef cattle [24]. They used Random Forest (RF), XGBoost, and RX to identify small subsets of biologically significant genes from a dataset of 16,423 genes. These gene subsets were then used to classify animals into high- and low-feed efficiency groups. Notably, RX identified the smallest subset of 117 genes, which outperformed those selected by traditional methods like t-test and edgeR, as well as other machine learning methods, in terms of classification accuracy. The accuracies in the trait prediction were evaluated with varying heritabilities and genetic architectures in Simmental beef cattle [25]. Their study, which analyzed 1301 samples with 671,990 SNPs, compared the performance of parametric methods (GBLUP and Bayes B) and two machine learning models: Cosine Kernel-based KRR (KcRR) and SVR. Their findings underscored the value of machine learning in refining trait predictions, particularly for complex traits such as live weight (LW), carcass weight (CW), and eye muscle area (EMA).

#### 8.3.3. Disease

Recent research has shown the potential of integrating genomic, metabolic, and machine learning approaches to enhance the prediction and diagnosis of health issues in dairy cattle, leading to more effective disease management and improved animal welfare [136]. Predictive potential of metabolic data was explored the in conjunction with milk performance records for subclinical ketosis risk in dairy cows [16]. The first five weeks’ worth of data from 218 dairy cows were the focus of their investigation. Using the β-hydroxybutyric acid concentration in milk as the target variable, the ANN models developed by the researchers achieved associations between observed and predicted values of up to 0.643. This study underscored the Possibility of merging the data to predict subclinical conditions in dairy cows, providing a foundation for early intervention strategies. A simulation study conducted to examine the efficacy of gBLUP and Random Forest (RF) in forecasting genetic risks for binary disease traits in dairy cows [26]. By calibrating the models with cow-specific genomic data, the study highlighted the strengths and limitations of both approaches, contributing to the ongoing exploration of machine learning in genetic disease prediction.

Further advancing the utilization of machine learning in cattle health management, The integration of Internet of Things (IoT) technology explored for diagnosing and predicting an extensive range of cattle diseases [27]. A dataset comprising 2000 samples from several cattle populations was assembled, each labeled according to the presence or absence of specific diseases such as milk fever, milk clots, watery milk, blisters, lameness, and various gastrointestinal and metabolic conditions. The primary objective was to evaluate the effectiveness of five machine learning models—Naïve Bayes Multinomial (NBM), lazy-IBk, Partial Tree (PART), Random Forest (RF), and Support Vector Machine (SVM)—in predicting cattle diseases. The results demonstrated the consistent superiority of the Random Forest model, which achieved the highest accuracy in disease prediction, reinforcing its potential as a robust tool for integrating IoT data in livestock health management.

## 9. The Concept of Phenomics and Its Advances in Animal Breeding

Phenomics, the high-dimensional acquisition of phenotypic data (Houle et al., 2010), represents a transformative shift in animal breeding, driven by advances in sensor technologies, machine learning, and data analytics [137]. These tools enable continuous and large-scale monitoring of novel and traditional traits, such as behavior, feed efficiency, and greenhouse gas emissions. Technologies like wearable sensors, computer vision, and spectroscopy allow previously inaccessible traits, such as social interactions or methane emissions, to be quantified. Phenomics addresses key societal demands, including animal welfare and sustainability, while enhancing understanding of the biological bases of traits. Integrating phenomics data with genetic evaluations holds the potential to redefine breeding programs by simultaneously optimizing productivity, resilience, and environmental efficiency.

However, phenomics faces challenges including complexity, heterogeneity, and high dimensionality of data from diverse sensors and time-dependent variables [138]. These issues necessitate advanced statistical methods, such as dimension reduction and penalization, to ensure robust predictions. Additionally, data standardization remains problematic due to the use of proprietary algorithms and non-comparable devices. Rural broadband limitations further hinder real-time data management. Despite these obstacles, phenomics is poised to revolutionize breeding through innovations in imputation techniques, deep learning for data integration, and reimagined breeding value models. With interdisciplinary collaboration, phenomics offers unparalleled opportunities to improve animal health, welfare, and productivity.

## 10. Challenges and Opportunities

The integration of genomic technologies into livestock breeding, particularly in developing countries, faces several significant challenges. One of the primary obstacles is the limited number and structure of reference populations, with most genotyped animals being females and numbering between 500 and 3000. The absence of artificially inseminating bulls further complicates the situation, making it difficult to implement comprehensive genomic selection programs. Additionally, the predominance of smallholder systems and the need to maintain indigenous breeds critical for biodiversity and sustainability add layers of complexity to breeding programs. However, these challenges also represent unique opportunities. The application of machine learning in genomic selection offers a promising solution to overcome the limitations of small reference populations. By improving the accuracy of breeding value predictions, ML algorithms can help optimize breeding programs even in resource-constrained settings. Furthermore, the preservation and incorporation of indigenous breeds into breeding strategies not only safeguard genetic diversity but also enhance the resilience of livestock to specific environmental conditions, thereby contributing to the sustainability of the agricultural industry. As the cost of genomic technologies continues to decrease, the potential for broader adoption in low- and middle-income countries increases, paving the way for more efficient and sustainable livestock production systems.

## 11. Future Prospects

The future of livestock breeding lies in the strategic integration of next-generation sequencing (NGS) technologies, machine learning (ML), and multi-omics data to enhance genetic gain and sustainability. As sequencing costs decline, large-scale genome-wide sequencing and cost-effective genotyping platforms will become more accessible, particularly in developing countries, enabling the study of indigenous and locally adapted breeds. Machine learning algorithms, including deep learning and artificial intelligence (AI), offer promising solutions to improve the accuracy of breeding value estimations, even in small reference populations. Future efforts should focus on developing robust ML models and computational frameworks to integrate multi-omics data, such as genomics, transcriptomics, and metabolomics, for precision breeding. This integration will accelerate genetic progress, reduce costs, and enable population-personalized breeding strategies. Additionally, capacity-building initiatives and knowledge transfer programs will be essential to ensure the adoption of these advanced technologies by breeders and farmers. Ethical considerations and sustainable breeding practices must also be prioritized to promote animal welfare and minimize environmental impacts. By leveraging these advancements, the livestock breeding community can drive genetic improvements, enhance productivity, and ensure the long-term sustainability of livestock production systems globally.

## 12. Conclusions

Advancements in livestock breeding have evolved from the foundational principles of Mendelian genetics to contemporary methods that incorporate cutting-edge technologies like genomic selection and machine learning. Initially, breeding relied on phenotypic selection based on observable traits, followed by quantitative genetics, which introduced statistical tools to enhance selection accuracy. The advent of molecular genetics brought DNA markers and marker-assisted selection (MAS), further refining breeding decisions. Today, genomic selection, supported by high-density SNP genotyping, has significantly improved the precision of breeding value estimates. The integration of big data and machine learning has further enhanced prediction accuracy, enabling more efficient and sustainable breeding practices. These technologies promise to revolutionize livestock breeding by addressing complex challenges like disease resistance and climate adaptability, ultimately leading to more resilient and productive livestock systems. However, ensuring the responsible application of these innovations, particularly for small-scale farmers, remains crucial for the future of sustainable animal agriculture.

## Figures and Tables

**Table 1:** Categories, focus areas, and key findings in the application of machine learning in livestock improvement.

Category	Reference	Focus	Models/Algorithms Used	Key Findings
**Milk Production**	[17]	Predicting yield and fertility traits in dairy cattle	ANN (Broyden-Fletcher-Goldfarb-Shanno algorithm)	R^2^ values: 0.444–0.989; strong predictive accuracy.
	[18]	Genomic prediction of residual feed intake in HF cattle	Self-trained vs. measured phenotypes	Reduced prediction accuracy as training set size increased. Maximum accuracy improvement: 5.9%.
	[19]	Predicting sire conception rate in Holstein cattle	Deep learning (MLP, CNN), Ensemble (RF, Gradient Boosting), Parametric (GBLUP, Bayes B)	Gradient Boosting achieved highest correlation (0.36), followed by Bayes B (0.34).
	[20]	Predicting pregnancy likelihood in dairy cows using AAM and on-farm data	Random Forest	Effective in reducing prediction errors by integrating diverse data sources.
**Beef Production**	[21]	Accuracy of genomic imputation tools in Angus cattle	Beagle, Fimpute, IMPUTE, AlphaImpute, AdaBoost-like	Beagle and Fimpute showed top accuracy (0.8677–0.9858).
	[22]	Predicting carcass traits in Hanwoo cattle	XGB, GBLUP	XGB excelled in carcass weight and marbling score; GBLUP performed better for backfat thickness and eye muscle area.
	[15]	Identifying SNPs for genomic relationship matrices in Brahman cattle	Random Forest, Gradient Boosting, XGBoost	Machine learning refined genomic selection by identifying key genetic markers.
	[23]	Forecasting genomic values in Simmental beef cattle	Adaboost.RT with SVR	Achieved 5.4–14.9% accuracy improvement over GBLUP.
	[24]	Classifying Nellore cattle into feed-efficiency groups	RF, XGBoost, RX	RX identified 117 significant genes with higher classification accuracy than traditional methods.
	[25]	Comparing heritabilities and genetic architectures in Simmental cattle	GBLUP, Bayes B, KcRR, SVR	Machine learning showed improved accuracy for traits like carcass weight and live weight.
**Disease**	[16]	Predicting subclinical ketosis risk in dairy cows	ANN	Predictive accuracy up to 0.643 for metabolic and milk performance data.
	[26]	Forecasting genetic risks for binary disease traits	gBLUP, RF	RF highlighted strengths in disease risk prediction; useful for exploring genetic risks.
	[27]	Integrating IoT data for cattle disease prediction	RF, NBM, lazy-IBk, PART, SVM	Random Forest showed highest accuracy for diagnosing diseases like milk fever, lameness, and metabolic conditions.

**Table 2:** A summary table of major QTL discoveries across different livestock species.

Species	Trait	Chromosome (QTL Location)	QTL IDs (Respective to CHR)	Key Findings	Reference
**Cattle**	Milk fat percentage	BTA14	**QTL ID: 10581**	DGAT1 on BTA14 influences milk fat content	[126]
**Cattle**	Growth rate, carcass traits	BTA16, BTA20, BTA21	**QTL ID: 1355, 1357, 1358**	QTLs linked to growth, meat quality	[127]
**Cattle**	Disease resistance (Clinical Mastitis)	BTA6, BTA2	**QTL ID: 137487, 283197**	QTLs on BTA6, CXCR1 gene on BTA2 associated with resistance against clinical mastitis	[128,129]
**Sheep**	Litter size, reproduction	OAR5, OARX	**QTL ID: 13837, 281743**	GDF9 and BMP15 mutations linked to prolificacy	[130]
**Sheep**	Greasy fleece weight	OAR6	**QTL ID: 238780**	Five FGF5 SNPs affect wool traits and growth in fine-wool sheep.	[131]
**Goat**	Body Weight	CHI25	**QTL ID: 255224**	MAPK3 genes associated with the QTL	[132]
**Pigs**	Growth, leanness, meat quality	SSC6, SSC11, SSC16	**QTL ID: 3650, 3211, 3214**	QTLs for backfat thickness, subcutaneous fat thickness, muscle mass	[133]
**Poultry**	Egg production, body weight	GGA1, GGAZ	**QTL ID: 177426, 19583**	QTLs affecting egg number, weight gain	[134,135]

## Data Availability

No data were used for the research described in the article.
